# Chronic Cough, Reflux, Postnasal Drip Syndrome, and the Otolaryngologist

**DOI:** 10.1155/2012/564852

**Published:** 2012-04-10

**Authors:** Deborah C. Sylvester, Petros D. Karkos, Casey Vaughan, James Johnston, Raghav C. Dwivedi, Helen Atkinson, Shah Kortequee

**Affiliations:** ^1^Department of Otolaryngology Head Neck Surgery, Bradford Royal Infirmary, Bradford BD96RJ, UK; ^2^Department of Otolaryngology Head Neck Surgery, Queen Alexandra Hospital, Portsmouth PO63LY, UK

## Abstract

*Objectives*. Chronic cough is a multifactorial symptom that requires multidisciplinary approach. Over the last years, general practitioners refer increasingly more chronic cough patients directly to the otolaryngologist. The aim of this paper is to highlight the issues in diagnosis and management of chronic cough patients from the otolaryngologist perspective. *Design*. Literature review. *Results*. Gastroesophageal reflux and postnasal drip syndrome remain one of the most common causes of chronic cough. Better diagnostic modalities, noninvasive tests, and high technology radiological and endoscopic innovations have made diagnosis of these difficult-to-treat patients relatively easier. Multidisciplinary assessment has also meant that at least some of these cases can be dealt with confidently in one stop clinics. *Conclusions*. As the number of referrals of chronic cough patients to an Ear Nose Throat Clinic increases, the otolaryngologist plays a pivotal role in managing these difficult cases.

## 1. Introduction

Chronic cough is a persistent and frustrating symptom for many adults and children and a frequent reason for primary or secondary care visits or referrals. This condition generates significant healthcare and economic cost and is associated with a spectrum of disorders across multiple medical specialties and can provide significant challenges for the involved physician or surgeon. Chronic cough is associated with deterioration in the quality of patients' lives. Associated symptoms and negative outcomes with this condition include loss of sleep, exhaustion, irritability, urinary incontinence, cough syncope, social disability, and inability to perform daily activities. Many patients experience chronic cough secondary to another medical condition, such as COPD, asthma, rhinosinusitis, Gastroesophageal reflux syndrome (GERD), postnasal drip syndrome (PNDS), or unknown etiology. GERD is thought to be the most common cause of chronic cough in a nonsmoker nonasthmatic individual.

Thorough assessment of a patient with a chronic cough relies on a multidisciplinary approach. The otolaryngologist should be familiar with the diagnostic algorithm of chronic cough patients and should work closely with the gastroenterologist and the pulmonologist, ideally in “cough clinics,” to confidently diagnose and treat these patients.

### 1.1. Reflux and Chronic Cough

Chronic nonspecific cough, defined as a nonproductive cough in the absence of identifiable respiratory disease or known cause [1] persisting for more than three to eight weeks [2], poses a significant burden to healthcare costs and considerably impairs quality of life. Gastroesophageal reflux disease (GERD) represents one of the three main causes of chronic cough (along with asthma and upper airways cough/postnasal drip syndrome), implicated in up to 41% of chronic cough patients [3]. The clinical features of GERD-related cough include heartburn, regurgitation, and/or worsening of cough after foods or medications known to decrease lower esophageal sphincter-pressure, with extraesophageal manifestations such as hoarseness, wheezing, sore throat, chest pain, and globus also described.

Whilst classic GERD symptoms are present in 6–10% of chronic cough patients, GERD is clinically silent in up to 75% of patients with GERD-related cough [4]. Diagnosis of GERD is frequently based on the clinical responses of cough to antireflux therapy rather than on objective assessments of GERD *per se*. Furthermore, an increased understanding of the pathophysiology of GERD and in particular the specific phenomenon of laryngopharyngeal reflux (LPR), has highlighted the complexity of this condition, with the need for individual patients assessment and tailoring of therapy becoming apparent.

Coughing may be provoked by reflux via a number of mechanisms. The regurgitation of gastric contents into the laryngopharynx can cause mechanical or pH-sensitive stimulation, with chronic inflammation leading to the sensitisation of peripheral nerves mediating cough [5]. This may have an acid or nonacid (namely, bile and pepsin) basis. Adhami et al. [6] demonstrated that bile can injure the laryngeal epithelium but only in an acidic environment, and furthermore, Sasaki et al. [7] were able to demonstrate histological laryngeal injury in a rat model following bile exposure in neutral environments. Pepsin, the principal proteolytic enzyme of the stomach, is predominantly active in acidic pH and has been shown to cause laryngeal injury in this state [6]; however proteolytic activity is still present up to pH 7 and can be reactivated [8]. Johnston et al. [8] found the presence of pepsin in the larynx of patients with the clinical diagnosis of LPR but not in controls and in these same patients, pepsin was absent in their esophageal epithelium [9]. It has been suggested that coughing can also be induced by “micro” or “silent” aspiration, caused by the direct activation of tracheo-bronchial receptors by reflux entering the airway. Distal esophageal reflux may also induce coughing through vagal stimulation known as the oesophago-bronchial reflex [10], Ing et al. [11], demonstrating that infusing acid into the oesophagus of chronic cough patients increases coughing. Additionally whilst infusion of acid (compared to saline) into the oesophagus of those GERD patients without chronic cough had no effect, a sensitised cough reflex to capsaicin was seen in those GERD patients with chronic cough [12].

An alternative pathophysiology is that coughing can in fact be the causation in reflux: increased intra-abdominal pressure during strenuous coughing episodes negatively impacting the lower esophageal sphincter, possibly by way of a positive feedback loop [13]. 

As discussed above, reflux associated cough can be a laryngopharyngeal or distal esophageal phenomena. LPR has distinct features, as first identified by Koufman and colleagues. In a combined reported series of 899 patients, throat clearing was a complaint of 87% of LPR patients versus 3% of those with GERD, while only 20% of LPR patients complained of heartburn versus 83% in the GERD group [5]. Differences in body mass index (BMI) between GERD and LPR patients have also been highlighted; in a retrospective study of 500 patients attending for pH probe studies, the mean BMI of isolated LPR patients was 25.9 compared to 28.3 for those with GERD [14]. 

Identifying GERD as the cause of a chronic cough can be challenging. Esophageal pH testing can demonstrate an increased number of reflux events, prolonged exposure of the esophageal mucosa to reflux, or more convincingly a significant temporal association between reflux events and cough. Although esophageal pH testing has a sensitivity of approximately 90% for the evaluation of chronic cough, specificity ranges from 66% to 100% [15, 16]. Additional diagnostic tests include inhaled tussigenic challenges, endoscopy, examining bronchoalveolar lavage fluid, and/or sputum for lipid laden macrophages, barium swallow, Bernstein test, radioisotope scintiscan, and radionucleotide emptying studies with solids [17]. Examination of the larynx may reveal evidence of LPR: key examination findings being vocal cord oedema and erythema as well as medial vocal cord erythema [18, 19]. Findings should, however, be taken in context: Hicks and colleagues finding that almost 80% of study participants had a least one reflux-attributable finding on laryngoscopy when 100 healthy volunteers were examined [20].

### 1.2. Treatment of GERD-Associated Cough

Patient counselling is essential in reducing GERD and related LPR. Dietary advice includes the avoidance of a high-fat diet and losing weight if obese, avoiding eating two hours before bedtime and refraining from caffeine, carbonated drinks, alcohol, and citrus products [21]. Patients should also be asked to refrain from smoking and elevate the head of the bed by 15 cm. Some medications are associated with increase GERD, namely, anticholinergics, beta-agonists, bisphosphonates, calcium-channel blocker, corticosteroids, benzodiazepines, oestrogens, opiates, progesterone, prostaglandins, and theophylline [17]. Other recommendations include nasal continuous positive airway pressure if obstructive sleep apnoea is present [22] and avoiding exercise that may increase intra-abdominal pressure [23].

Proton pump inhibitors (PPIs) have commonly been the mainstay empirical treatment for GERD-related cough. Given the difficulty in clearly diagnosing this condition, Irwin [21] has described the clinical profile of such patients in whom empirical therapy should be considered; those not exposed to environmental irritants, not a present smoker, not on an ACE inhibitor, with a normal/stable chest radiograph, and in whom symptomatic asthma, upper airways cough syndrome, and nonasthmatic eosinophilic bronchitis has been ruled out. The use of empirical therapy has, however, been questioned. In a meta-analysis of 5 randomised controlled trials on GERD treatment for cough in adults and children without primary lung disease, Chang et al. [24] found that there was no difference in cough resolution for patients who received a placebo versus a PPI (OR 0.24 (95% CI 0.04 to 1.27). There was, however, a significant difference in secondary outcomes of mean cough score (mean difference of −0.51 (−1.02 to 0.01)) and change in cough score (−0.29 (−0.62 to 0.04)) at the end of the trial. This led the authors to conclude that the use of PPI had “some effect in some adults.” More recently, a Cochrane Database Systematic review by Chang and colleagues [2] including 9 randomised controlled trials of PPIs for adults with chronic cough found that using intention-to-treat, pooled data from studies resulted in no significant difference between treatment and placebo in total resolution of cough (OR 0.46; 95% CI 0.19 to 1.15 no overall significant improvement in cough outcomes (end of trial or change in cough scores). There was, however, a significant improvement in cough scores at end of intervention (two to three months) in those receiving PPI (standardised mean difference −0.41; 95% CI −0.75 to −0.07) using generic inverse variance analysis on cross-over trials. The authors were unable to conclude definitely that GERD treatment with PPIs is universally beneficial for cough associated with GERD. Despite the current lack of evidence for definite treatment of empiric treatment, published guidelines from the ACCP [21] and BTS [25] suggest that PPI therapy should be commenced, for example, omeprazole 20–40 mg twice daily or equivalent taken before meals for at least 8 weeks [25]. 

Of particular interest in nonacidic refux, medications such as Gaviscon or Gaviscon Advance, which act by forming a raft or physical barrier to reflux present a supplementary or even alternative treatment option. McGlashan et al. [26] conducted a randomised controlled trial of Gaviscon Advance in 49 patients with a diagnosis LPR (based on the reflux symptom index (RSI) and the reflux findings score (RFS)). Patients were assessed pretreatment and at 2, 4, and 6 months after treatment. Significant differences in the mean (SD) between treatment and control were observed for RSI at the 2-month (11.2 (7.0) versus 16.8 (6.4), *P* = 0.005) and 6-month (11.2 (8.1) versus 18.3 (9.4), *P* = 0.008) assessments and for RFS at the 6-month (7.1 (2.8) versus 9.5 (3.4), *P* = 0.005) assessment. The details of the cough component of the RSI were not, however, detailed further in the report.

Gastroesophageal dysmotility has been implicated in the pathophysiology of GERD via abnormalities of delayed gastric emptying and reduced pressure or inappropriate transient relaxation of the lower esophageal sphincter [27]. Several prokinetic agents (e.g., bethanechol, metoclopramide, domperidone, cisapride, and macrolides such as erythromycin) can stimulate gastrointestinal motility and have, therefore, been proposed as useful adjuncts to antireflux medication. The evidence base for this lies only in unblinded, uncontrolled studies, where, when in combination PPIs for treating GERD-associated cough, cough or hoarseness improved by 70% to 100% [10, 28–31]. In the recent Cochrane review, Chang et al. [2] found insufficient data to evaluate the evidence for the use of prokinetic agents in chronic cough. However, interestingly in 56 patients diagnosed with GERD-related cough, 24 responded to a PPI alone; however, 18 of the remaining patients improved with the addition of metoclopramide or cisapride [32]. These drugs, however, may have significant side-effects: erythromycin, for example, often causes nausea and abdominal pain and cisapride was withdrawn from the US market due to safety concerns.

Although surgery is more traditionally used to treat the more typical reflux symptoms, it may be of some value in the management of reflux-related cough. Studies relating to the outcomes of surgical treatment of GERD are, however, of questionable value as they suffer from lack of controls and blinding, use differing postoperative evaluation criteria, and are typically based on a highly selective group of patients. Kaufman and colleagues [33] reported their long-term (mean 53 months) outcomes of 128 patients treated with laparoscopic antireflux surgery. Cough and hoarseness was improved in 65% to 75% of cases compared to heartburn and regurgitation in over 90% of subjects. In their review of treatment options for GERD-related cough, Chandra and Harding [17] summarised the finding of 9 prospective studies of surgical management, reporting that 586 of 689 surgically treated patients had a “significant cough response.”

GERD remains one of the leading causes of chronic cough; however, the difficulty in diagnosing this condition, especially as “classic” reflux symptoms are often absent means that it can be overlooked. The mainstay of treatment, for now, remains as lifestyle modification, dietary advice, and medical therapy. The role of traditional empirical treatment with PPIs is questionable, with evidence from randomised trials implying that there is some benefit in the right patient group. Identifying this patient group is, therefore, imperative: careful clinical history taking and laryngeal evaluation along with objective reflux assessment being key. The use of alginate preparations seems to be quite popular in the last few years, although evidence is currently lacking. Surgical management, although not as useful for cough symptomatology as for classic symptoms also has a role in patients resistant to medical therapy.

## 2. Postnasal Drip Syndrome (or Upper Airway Cough Syndrome)

Postnasal drip (PND) or catarrh is the drainage of secretions from the nose or paranasal sinuses into the pharynx. Clinically, the diagnosis of PND syndrome (PNDS) is very vague, made on history and examination and relies on the reporting of the patient of this sensation of something “dripping down the throat,” rhinorrhoea and constant throat clearing [34]. Nasendoscopy revealing rhinitis and mucopurulent secretions is suggestive, although not diagnostic. The issues when attempting to diagnose PNDS, is that there are no objective sensitive or specific tests and no way to quantify the amount of catarrh or to prove that it is directly responsible for causing cough. PNDS is associated with very nonspecific symptoms and a definitive diagnosis of PND-induced cough cannot be made from the history and examination findings alone.

The differential diagnosis of PNDS-induced cough includes all other causes of rhinitis including, allergic rhinitis, perennial nonallergic rhinitis, bacterial sinusitis, allergic fungal sinusitis, rhinitis due to anatomic sinonasal abnormalities, rhinitis due to physical or chemical irritants, occupational rhinitis, rhinitis medicamentosa, and rhinitis of pregnancy.

Another issue when attempting to diagnose PNDS-induced cough is that GERD is often associated with a high prevalence of upper respiratory symptoms and therefore can either coexist or mimic PNDS [35]. The introduction of the-more widely accepted in Americas-term of “Upper Airway Cough Syndrome” (UACS) was made based on the need to answer the question whether “the conditions listed above actually produce cough through a final common pathway of PND or whether, in fact, in some circumstances they cause irritation or inflammation of upper airway structures that directly stimulate cough receptors and produce cough independently of or in addition to any associated PND” [35]. 

It is obvious that because there are no objective tests for diagnosing PND, treatment is often based on the specific disease that is present. For example, avoidance of specific allergens after allergy testing has been done, nasal steroid treatment and antihistamines, treatment of concomitant infection, and correction of any associated sinonasal anatomical abnormalities can have an indirect effect on the management of PND-induced cough.

The American College of Chest Physicians recommends an empiric trial of therapy for UACS because improvement or resolution of cough in response to specific treatment is the pivotal factor in confirming the diagnosis of UACS as a cause of cough. That should especially be the case if no specific cause can be elicited from the history and examination [36]. 

A usual empiric therapy involves a first generation antihistamine/decongestant. If a patient has resolution or partial resolution of cough, then UACS is considered to have been a cause of cough and antihistamine therapy is continued. Marked improvement or resolution of cough may take several weeks and occasionally as long as a few months [37]. 

If there is no response with a first-generation antihistamine, then the patient should undergo sinus imaging. Chronic sinusitis causes a productive cough or can be clinically silent, in that the cough can be nonproductive, and none of the typical findings associated with acute sinusitis may be present [36, 38]. 

Allergy skin testing, measurement of serum Ig levels to see whether (acquired) hypogammaglobulinemia is present, evaluation of the patient's home and workplace if there is a potential environmental cause for persistent upper airway symptom are all reasonable diagnostic strategies especially if there is lack of response to sinusitis treatment. If nasendoscopy reveals nasal polyps, in the absence of any contraindication, the patient should undergo a standard aspirin challenge. If the results of the challenge are positive, the patient should undergo desensitization, followed by the consideration of chronic aspirin therapy unless it is contraindicated.

## 3. Conclusions

Chronic cough can be associated with many diseases that often overlap more than one medical specialty. A detailed assessment of the patient with chronic cough relies on a multidisciplinary approach and close cooperation between pulmonary medicine, gastroenterology, and otolaryngology. Gastrooesophageal reflux and postnasal drip syndrome account for a significant number of cases of chronic nonproductive cough seen in otolaryngology practice. Each may, alone or in combinationcontribute to cough even when clinically silent, and failure to recognise their contribution may lead to unsuccessful treatment. Many of these patients are notoriously difficult to diagnose and treat but the literature suggests that a systematic and thorough approach in a multidisciplinary setting can lead to successful diagnosis and treatment in the majority of patients.
